# Ultrahigh Electrocatalytic Conversion of Methane at Room Temperature

**DOI:** 10.1002/advs.201700379

**Published:** 2017-09-11

**Authors:** Ming Ma, Bing Jun Jin, Ping Li, Myung Sun Jung, Jin Il Kim, Yoonjun Cho, Sungsoon Kim, Jun Hyuk Moon, Jong Hyeok Park

**Affiliations:** ^1^ Department of Chemical and Biomolecular Engineering Yonsei University 50 Yonsei‐ro Seodaemun‐gu Seoul 120‐749 Republic of Korea; ^2^ SKKU Advanced Institute of Nano Technology (SAINT) Sungkyunkwan University Chunchun‐dong Suwon 440‐746 Republic of Korea; ^3^ Department of Chemical and Biomolecular Engineering Sogang University 1 Sinsu‐dong Mapo‐gu Seoul 121‐742 Republic of Korea

**Keywords:** 1‐propanol, 2‐propanol, electrochemical, methane oxidation, ZrO_2_/Co_3_O_4_

## Abstract

Due to the greenhouse effect, enormous efforts are done for carbon dioxide reduction. By contrast, more attention should be paid for the methane oxidation and conversion, which can help the effective utilization of methane without emission. However, methane conversion and utilization under ambient conditions remains a challenge. Here, this study designs a Co_3_O_4_/ZrO_2_ nanocomposite for the electrochemical oxidation of methane gas using a carbonate electrolyte at room temperature. Co_3_O_4_ activated the highly efficient oxidation of methane under mild electric energy with the help of carbonate as an oxidant, which is delivered by ZrO_2_. Based on the experimental results, acetaldehyde is the key intermediate product. Subsequent nucleophilic addition and free radical addition reactions accounted for the generation of 2‐propanol and 1‐propanol, respectively. Surprisingly, this work achieves a production efficiency of over 60% in the conversion of methane to produce these long‐term stable products. The as‐proposed regional electrochemical methane oxidation provides a new pathway for the synthesis of higher alcohols with high production efficiencies under ambient conditions.

## 1 Introduction

Methane (CH_4_) is the primary component of natural gas, which constitutes 21.4% of the total primary energy sources in the world,^1^ and is widely used as an important fuel in both industrial chemical process and human daily life. Compared with other fossil fuels, such as oil and coal, the combustion of natural gas provides lower carbon dioxide (CO_2_) emissions,^2^ making natural gas a suitable alternative transitional energy source until carbon‐free energy sources are sufficiently mature to be deployed.^3^ However, the emission of CH_4_ gas has long been ignored and regarded as a trivial matter,^4^ even though its effect as a greenhouse gas is over 30 times more potent than that of CO_2_.^5^ In particular, global warming and the exploitation of shale gas aggravate this emission. Recently, more attention has been given to the negative impact of CH_4_ emissions due to increasing environmental pollution and climate change.^6^, ^7^, ^8^ Hence, efforts have focused on the conversion of atmospheric CH_4_ into an equimolar amount of CO_2_ through thermocatalysis or photocatalysis. However, conventional CH_4_ conversion processes still suffer from various drawbacks, including the use of precious metal catalysts, high reaction temperatures that are far from ambient conditions and extremely low conversion efficiencies.^9^, ^10^, ^11^ In this regard, the oxidation and conversion of CH_4_ to liquid alcohols, such as methanol, ethanol, and propanol, is much more economical and energy‐efficient. Among liquid alcohols, higher alcohols with high energy densities have wide applications in fabricating commodity chemicals.^12^

Methanol, as a product of CH_4_ conversion, has been widely investigated. Currently, the syngas reaction is the main route for the industrial production of methanol, whereby syngas is produced through CH_4_ steam reforming. The two reactions for CH_4_ conversion to methanol are given in Equations (1) and (2), 13, 14

(1) $${\mathrm{CH}}_{4}(g)\;+\;H_{2}O(g)\;\overset{\mathrm{Ni}}{\to }\;\mathrm{CO}(g)\;+\;3H_{2}(g)\;\Delta H_{\mathrm{298}}\;=\;\mathrm{49}.3\;\mathrm{kcal}\;{\mathrm{mol}}^{-1}$$

(2) $$\mathrm{CO}(g)\;+\;2H_{2}(g)\;\overset{{\mathrm{Cu/ZnO/Al}}_{2}^{}\;O_{3}^{}}{\to }\;{\mathrm{CH}}_{3}\mathrm{OH}(g)\;\Delta H_{\mathrm{298}}\;=\;-\mathrm{21}.7\;\mathrm{kcal}\;{\mathrm{mol}}^{-1}$$

From the equations, the initial reaction in syngas reforming requires more energy than that released from the second reaction, illustrating that additional energy input is necessary for the conversion of CH_4_ to methanol. Some scientists have employed a class of bacteria known as methanotrophs with methane mono‐oxygenases to convert CH_4_ into methanol under ambient conditions using oxygen.^15^ The methods mentioned above require complex processes, extra energy consumption, or enzyme cultivation and critical control of the conditions.^13^, ^16^ In fact, the direct oxidation of CH_4_ by oxygen gas (O_2_) is always accompanied by substantial overoxidation, which is both kinetically and thermodynamically favorable.^13^ The conversion of CH_4_ to methanol with O_2_ is triggered by energy input and proceeds spontaneously with a substantial release of energy, which makes it exergonic for the further oxidation of methanol to formaldehyde, formic acid, carbon monoxide (CO), and CO_2_. Controlling CH_4_ oxidation to obtain methanol is extremely difficult. Thus, compared with the route of inhibiting CH_4_ overoxidation to obtain methanol, the control of further oxidation processes under mild conditions may enable the production of more useful higher alcohols, such as propanol. Electrochemical oxidation in aqueous electrolyte has been demonstrated to be a suitable method for the conversion of CH_4_ or other volatile organic compounds (VOCs) at low temperature using simple reaction instruments.^17^, ^18^, ^19^

The C—H bonds in CH_4_ have a high dissociation energy of 104 kcal mol^−1^, which makes CH_4_ extremely inert. When O_2_ is used as an oxidizing agent for CH_4_ oxidation, the reaction of triplet O_2_ with singlet CH_4_ to form singlet methanol is a spin‐forbidden process.^13^ Thus, protons in CH_4_ are not expected to be readily abstracted by O_2_ under mild conditions, such as low temperature. Therefore, other oxidizing agents are necessary to replace O_2_. In conventional alkaline electrochemical systems, which are always performed at room temperature, hydroxide (HO^−^) generally functions as the oxidizing agent. However, hydroxide has previously been demonstrated to have negligible activity for abstracting protons from CH_4_ under mild conditions.^20^ By contrast, carbonate (CO_3_^2−^) oxidizes species by donating a charged oxygen atom accompanied by CO_2_ release, resulting in a large enthalpy of reaction and favorable oxidation kinetics.^21^, ^22^, ^23^ Therefore, CO_3_^2−^ may be an attractive alternative to OH^−^ for alkaline electrochemical reactions. In addition, to realize the donation of an oxidizing agent from CO_3_^2−^, zirconia (ZrO_2_) should be employed to facilitate CO_3_^2−^ adsorption because of its surface Lewis acid sites and electron‐accepting capabilities.^24^, ^25^ Based on the above results, Mustain's group selected nickel oxide (NiO) as the catalyst and prepared NiO/ZrO_2_ for the electrochemical oxidation of CH_4_ with CO_3_^2−^ as the oxidizing agent.^26^ However, from their results, NiO did not exhibit satisfactory selectivity for CH_4_ oxidation, and the reaction mechanism was also unclear. Thus, more efficient catalysts with high activity and selectivity should be employed.

**Scheme 1:**
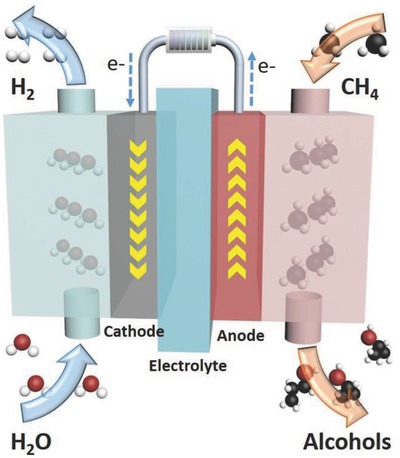
The reaction process of electrochemical oxidation of methane gas.

Among metal oxide catalysts, cobalt oxide (Co_3_O_4_) has been demonstrated to be one of the most efficient catalysts for the oxidation of VOCs.^27^ In addition, Co_3_O_4_ has a strong surface adsorption capacity for formaldehyde.^28^ According to theoretical observations of the reduction of CO_2_ on transition metal surfaces,^29^ formaldehyde tends to be oxidized to CO. However, when adsorbed on the surface of Co_3_O_4_, formaldehyde, converted from methanol, may be more active for further additional reactions to higher alcohols, illustrating the regional selectivity of Co_3_O_4_ for CH_4_ oxidation. Thus, we designed a Co_3_O_4_/ZrO_2_ composite for the electrochemical oxidation of CH_4_, using CO_3_^2−^ as the oxidizing agent source, that resulted in high selectivity for the production of 1‐propoanl and 2‐propanol with over 60% production efficiency. The reaction process is shown in **Scheme** **1**.

## 2 Results and Discussion

### 2.1 Fabrication and Characterization of the Co_3_O_4_/ZrO_2_ Nanocomposite

**Figure 1:**
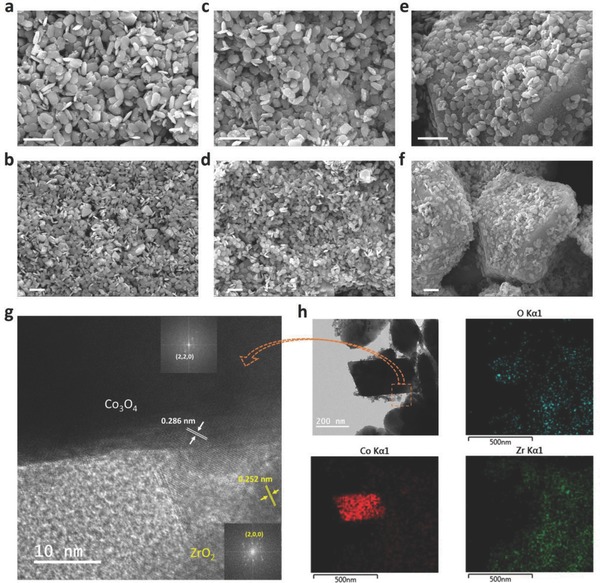
Morphologies of the ZrO_2_/Co_3_O_4_ nanocomposites with different ratios. SEM images of the a,b) 1–2 ZrO_2_/Co_3_O_4_, c,d) 1–4 ZrO_2_/Co_3_O_4_, and e,f) 1–6 ZrO_2_/Co_3_O_4_ samples. The scale bars in (a)–(f) are 1 µm. g) HR‐TEM image and h) TEM image with elemental mappings of the 1–4 ZrO_2_/Co_3_O_4_ sample. The inserts are fast‐Fourier transformation patterns.

Coprecipitation and hydrothermal methods were employed to fabricate the Co_3_O_4_/ZrO_2_ nanocomposite (for details, see the Experimental Section). Scanning electron microscopy (SEM) was used to observe the morphology of different samples fabricated using different ZrO_2_/Co_3_O_4_ ratios, denoted 1–2 ZrO_2_/Co_3_O_4_, 1–4 ZrO_2_/Co_3_O_4_, and 1–6 ZrO_2_/Co_3_O_4_, as shown in **Figure** **1**. In all samples, oval‐shaped ZrO_2_ nanoparticles with uniform sizes were formed and were adsorbed on the surface of the Co_3_O_4_ plates. Upon increasing the amount of Co_3_O_4_, the particle size of Co_3_O_4_ gradually increased until it reached bulk (Figure 1; and Figure S1, Supporting Information), which may affect the catalytic properties of the ZrO_2_/Co_3_O_4_ nanocomposite. Pure Co_3_O_4_ powder was also prepared for comparison (Figure S2, Supporting Information) and showed large particles that were more than 10 µm in size. The size of the Co_3_O_4_ plates can be controlled through coprecipitation with ZrO_2_. The elemental ratios of Zr/Co for all samples were obtained through EDS (energy‐dispersive X‐ray spectroscopy) measurement (Table S1 and Figure S3, Supporting Information). The Zr/Co ratio decreased upon increasing the amount of Co precursor. In the 1–6 ZrO_2_/Co_3_O_4_ sample, big Co_3_O_4_ plates were surrounded by small ZrO_2_ particles, which may have caused the sudden decrease in the Zr/Co ratio. The EDS measurement was focused on the surface of the samples, which would be affected much by the surface state of the materials. Thus, the ratios showed different value when compared to the stoichiometric ratio of elements calculated from the reactants described in the Experimental Section. In order to obtain the stoichiometric ratio of Zr/Co, ICP‐OES (inductively coupled plasma optical emission spectrometry) measurement was conducted, shown in Table S1 (Supporting Information). The 1–4 ZrO_2_/Co_3_O_4_ sample exhibited relatively small particle sizes and a suitable amount of Co_3_O_4_ and showed the best electrochemical catalytic performance for CH_4_ oxidation, which will be discussed next.

**Figure 2:**
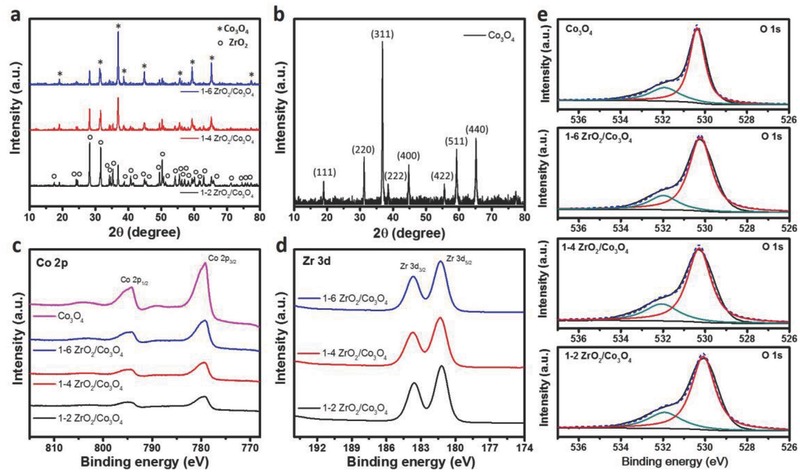
Characteristics of the ZrO_2_/Co_3_O_4_ nanocomposite. XRD spectra of a) the 1–2 ZrO_2_/Co_3_O_4_, 1–4 ZrO_2_/Co_3_O_4_, and 1–6 ZrO_2_/Co_3_O_4_ samples and b) pure Co_3_O_4_. c) Co 2p XPS signals of pure Co_3_O_4_ and ZrO_2_/Co_3_O_4_ samples with different ratios of 1–2, 1–4, and 1–6. d) Zr 3d XPS signals of ZrO_2_/Co_3_O_4_ samples with different ratios of 1–2, 1–4, and 1–6. e) Deconvoluted O 1s XPS signals of pure Co_3_O_4_ and ZrO_2_/Co_3_O_4_ samples with different ratios of 1–2, 1–4, and 1–6.

For further examination of ZrO_2_ and Co_3_O_4_, 1–4 ZrO_2_/Co_3_O_4_ was examined by transmission electron microscopy (TEM) (Figure 1g,h). In the TEM image (Figure 1h), the Co_3_O_4_ plate can be easily distinguished from the surrounding ZrO_2_ nanoparticles. From the high‐resolution TEM (HR‐TEM) image (Figure 1g), the lattice constant of ZrO_2_ was found to be 0.252 nm, corresponding to the (002) facet, and that of Co_3_O_4_ was 0.238 nm, corresponding to the (022) facet. In addition, elemental mapping was performed for Co, Zr, and O atoms (Figure 1h). From the elemental distribution, the Co_3_O_4_ plates and ZrO_2_ nanoparticles were confirmed to have different structures. From the SEM and TEM images, well physical connection between ZrO_2_ and Co_3_O_4_ could be demonstrated, which may lead to synergistic effects in the electrochemical oxidation of CH_4_. In addition, from the X‐ray diffraction (XRD) and X‐ray photoelectron spectroscopy (XPS) spectra (**Figure** **2**), peaks of each sample showed almost same position without shifting, illustrating no obvious chemical bondings can be observed between ZrO_2_ and Co_3_O_4_.

The crystalline structures of the ZrO_2_/Co_3_O_4_ composites with different ratios were analyzed by powder XRD using Cu Kα radiation and then compared with that of pure Co_3_O_4_ (Figure 2a,b). The diffraction peaks of ZrO_2_ corresponded to the monoclinic phase (JCPDS No. 37‐1484), and the peaks of Co_3_O_4_ were indexed to the cubic structure (JCPDS No. 42‐1467). In the XRD patterns, the typical (001), (100), (011), (−111), and (022) planes of ZrO_2_ were observed at ≈17.5°, 24.2°, 24.6°, 28.3°, and 50.3°. All related peaks gradually decreased in intensity as the amount of Co_3_O_4_ increased. The typical (111), (311), and (440) planes of Co_3_O_4_ were observed at ≈19.0°, 36.9°, and 65.2°, which also showed the same change in peak intensity with an increase in ZrO_2_.

The abovementioned data explained the microscopic and crystalline structures of the ZrO_2_/Co_3_O_4_ nanocomposite. Therefore, we next set out to determine the surface state, which is responsible for the unique regional selectivity of CH_4_ oxidation, by XPS, as shown in Figure 2c–e. XPS spectra of the Co 2p (Figure 2c), Zr 3d (Figure 2d), and O 1s (Figure 2e) core levels, along with a survey scan (Figure S4a, Supporting Information), were obtained for the samples with different ZrO_2_/Co_3_O_4_ ratios. The binding energies of the Co and Zr signals did not greatly shift upon changing the component ratio. However, the intensity of the Co signals clearly changed as the amount of Co decreased. The Zr signals also changed, but not as much as the Co signals, which may be due to ZrO_2_ being on top of the Co_3_O_4_ surface. Additionally, the O 1s spectroscopic signals were affected. The O 1s peaks at ≈530 and 532 eV are ascribed to lattice oxygen and nonuniform surface sites, respectively.^30^ The peak at ≈532 eV is related to defect sites on the surface, such as chemisorbed or dissociated oxygen or hydroxyl species.^31^ The surface of Co_3_O_4_ is known to be readily covered with a monolayer of negatively charged chemisorbed oxygen.^28^ In this case, the peak at ≈532 eV in the O 1s spectrum of pure Co_3_O_4_, shown in Figure 2e, can be clearly deconvoluted to demonstrate the surface adsorption ability of Co_3_O_4_. In addition, after coprecipitation with ZrO_2_, the O 1s signals of all ZrO_2_/Co_3_O_4_ composites showed enhanced peaks at ≈532 eV, which can be observed more clearly in the overlapping plots in Figure S4b (Supporting Information). This result shows the surface electron accepting capabilities of ZrO_2_, as reported previously,^24^, ^25^ indicating its strong adsorption of CO_3_^2−^ electrolyte during the electrochemical oxidation of methane.

### 2.2 Electrochemical Performance for CH_4_ Oxidation

To measure the electrochemical performance of CH_4_ oxidation, a glassy carbon disc was employed to load the catalyst, forming the working electrode. Details of the preparation and measurement process are provided in the Experimental Section. As mentioned above, NiO is not an efficient catalyst for CH_4_ oxidation. Thus, we prepared a ZrO_2_/NiO composite and compared its catalytic ability with that of ZrO_2_/Co_3_O_4_, as shown in Figure S5 (Supporting Information). In the measurement of the current–voltage (*J*–*V*) curves and Nyquist plots (for impedance analysis), argon (Ar) saturation was used to analyze the water oxidation and CH_4_ saturation was used to analyze the CH_4_ oxidation competing with water oxidation. Additional anodic activity under CH_4_ saturation indicated that both NiO and Co_3_O_4_ have CH_4_ oxidizing activity. However, CH_4_ oxidation performed better on the surface of Co_3_O_4_ than NiO. The same results were observed via electrochemical impedance spectroscopy (EIS) (Figure S5b,d, Supporting Information), which illustrated the superior activity of Co_3_O_4_ for CH_4_ electrochemical oxidation.

**Figure 3:**
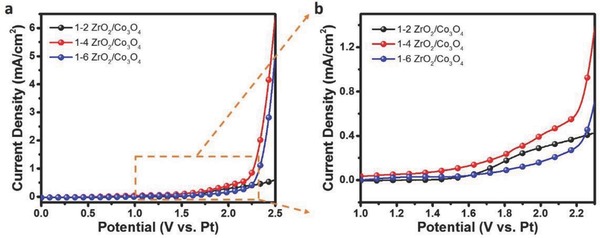
Electrochemical performance of CH_4_ oxidation. a) *J*–*V* curves and b) magnified curves of the ZrO_2_/Co_3_O_4_ samples with different ratios of 1–2, 1–4, and 1–6.

**Figure** **3** shows the linear sweep voltammetry (LSV) curves of the ZrO_2_/Co_3_O_4_ samples with different ratios in CH_4_‐saturated carbonate electrolyte. The 1–4 ZrO_2_/Co_3_O_4_ sample showed the highest electrochemical current density for CH_4_ oxidation, which agrees with the sample morphology results after adjusting the component ratio, as 1–4 ZrO_2_/Co_3_O_4_ had a relatively suitable size and amount of Co_3_O_4_. The current density of the 1–2 ZrO_2_/Co_3_O_4_ sample was relatively low even after increasing the potential to a high value, which illustrated that a low amount of Co_3_O_4_ provided poor catalytic activity in CH_4_ oxidation. By contrast, ZrO_2_ showed extremely weak oxidizing activity under both H_2_O and CH_4_ saturation due to its high bandgap of more than 5 eV.^32^ The 1–6 ZrO_2_/Co_3_O_4_ sample showed the same *J*–*V* curve trend as the 1–4 ZrO_2_/Co_3_O_4_ sample but a lower current density value, illustrating the weaker surface adsorption ability toward the carbonate electrolyte due to the lower amount of ZrO_2_. In addition, *J*–*V* curves of the optimized sample in Ar‐ and CH_4_‐saturated electrolyte were recorded to demonstrate its ability for CH_4_ oxidation, as shown in Figure S6a (Supporting Information). In addition, pure Co_3_O_4_ powder was also prepared to examine the electrochemical oxidation of CH_4_ (Figure S6b, Supporting Information). Unfortunately, the Co_3_O_4_ sample without ZrO_2_, which aids the adsorption of oxygen donors, showed no additional anodic activity in CH_4_‐saturated electrolyte and even worse performance, which confirmed that the outstanding electrochemical oxidation of CH_4_ resulted from the synergistic effects of ZrO_2_ and Co_3_O_4_, i.e., carbonate adsorption and CH_4_ oxidation, respectively.

### 2.3 CH_4_ Conversion Measurement and Product Analysis

**Figure 4:**
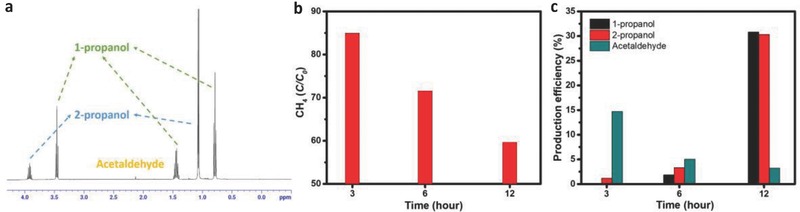
Product analysis and production efficiency. a) ^1^H‐NMR spectrum of the products after 12 h. b) The amount of CH_4_ remaining after electrochemical oxidation. c) Production efficiencies of the products of 1‐propanol, 2‐propanol, and acetaldehyde at different reaction times.

In the measurement of the CH_4_ conversion, the catalyst was loaded on carbon paper to form the anode, carbonate was the electrolyte and platinum foil was the counter electrode. The preparation and measurement processes are shown in the schematic diagram in Figure S7 (Supporting Information), and further details are provided in the Experimental Section. CH_4_ conversion using the Co_3_O_4_ catalyst was performed to obtain more stable and useful higher alcohols. Therefore, a sealed reactor instrument is more suitable for the conversion and for tracking the CH_4_ oxidation progress in real time. To determine the optimal potential for long reaction times, the difference in the current density of the optimized sample in Ar‐ and CH_4_‐saturated electrolyte (see Figure S6a, Supporting Information) was calculated, and the curves are provided in Figure S8 (Supporting Information). Based on the curves, 2.0 V was selected as the suitable potential for CH_4_ electrochemical oxidation to obtain a relatively high current density and low competition with water oxidation. The products were collected after reaction for 3, 6, and 12 h with vigorous stirring. *I*–*t* curves for 12 h measurement was shown in Figure S9 (Supporting Information). To identify the products, proton nuclear magnetic resonance (^1^H‐NMR) spectroscopy was performed. **Figure** **4**a shows the ^1^H‐NMR spectrum of the products obtained after 12 h of reaction. The main products were 1‐propanol and 2‐propanol.^33^, ^34^ However, by‐products, such as methanol, ethanol, acetaldehyde, and acetone, were also observed. The typical ^1^H‐NMR peak of methanol is located at ≈3.3–3.5 ppm,^34^ which may overlap with that of 1‐propanol. The ^1^H‐NMR peaks of ethanol appear at the same positions as the 2‐propanol peaks.^34^ However, the subpeak numbers of ethanol and 2‐propanol are different; thus, the product can be identified as 2‐propanol. However, small ethanol peaks with weak peak intensity may be obscured by the 2‐propanol peaks. The small peak at ≈2.2 ppm can be ascribed to acetaldehyde and acetone.^33^ Compared with the main products (1‐propanol and 2‐propanol), all the by‐products were observed in negligible quantities. In order to eliminate other influence factors, control experiment was conducted at the same condition for 12 h long‐term reaction except the presence of CH_4_. The ^1^H‐NMR result of the products from control experiment was shown in Figure S10a (Supporting Information). In addition, the pure carbonate electrolyte before reaction was also detected with ^1^H‐NMR spectrum for comparison (Figure S10b, Supporting Information). To confirm the amount of CH_4_ consumed and products generated, gas chromatography (GC) and GC‐mass spectrometry (MS) were performed. The amount of CH_4_ remaining in the reactor after 3, 6, and 12 h of reaction is shown in Figure 4b. In addition, the reference line of the amount of CH_4_, measured by GC, is shown in Figure S11 (Supporting Information). CH_4_ gas was mostly consumed, and the amount decreased gradually with the reaction time. After 12 h of reaction, almost 40% of the CH_4_ gas was converted. Meanwhile, the amount of various products measured by the GC‐MS system is shown in **Table** **1**.

**Table 1:** Concentrations of various products after the electrochemical oxidation of CH_4_ for 3, 6, and 12 h

Time [h]	Methanol [μg mL^−1^]	Formaldehyde [μg m L^−1^]	Ethanol [μg mL^−1^]	Acetaldehyde [μg mL^−1^]	1‐Propanol [μg mL^−1^]	2‐Propanol [μg mL^−1^]	Acetone [μg mL^−1^]
3	29.95	0.88	0	261.50	0.50	19.53	0
6	33.15	1.11	0.49	170.34	56.51	101.74	3.82
12	33.71	1.10	35.21	153.42	1336.12	1315.56	14.16

As described in Table 1, seven products were detected: methanol, formaldehyde, ethanol, acetaldehyde, 1‐propanol, 2‐propanol, and acetone. The amount of methanol and formaldehyde (products containing one carbon atom) did not greatly change with reaction time, illustrating a balance between generation and consumption. Thus, methanol and formaldehyde are the primary products of CH_4_ oxidization. As detailed in a previous study, formaldehyde should be the methanol oxidation product.^23^ After comparing the amount of ethanol and acetaldehyde (products containing two carbon atoms), acetaldehyde can be confirmed as the main product from the addition reaction of CH_4_ and formaldehyde. Moreover, the amount of acetaldehyde decreased with the reaction time, which illustrates that acetaldehyde plays a pivotal role in the production of 1‐propanol and 2‐propanol, indicating that 1‐propanol and 2‐propanol were converted from acetaldehyde. Then, after 12 h of reaction, 1‐propanol, and 2‐propanol were the main stable products of CH_4_ oxidation, which agrees with the ^1^H‐NMR results. The conversion efficiencies for acetaldehyde, 1‐propanol and 2‐propanol were calculated and are shown in Figure 4c. After 12 h of reaction, the main products, 1‐propanol and 2‐propanol, showed total production efficiency of over 60%.

### 2.4 Reaction Mechanism Analysis

As observed in the results in Table 1, acetaldehyde was the key product. The reactions involved in the formation of acetaldehyde are shown below, according to previous investigations^35^

(3) $${\mathrm{CH}}_{4}\;\overset{\mathrm{oxidant}}{\to }\;{\mathrm{CH}}_{3}\mathrm{OH}\;\overset{\mathrm{oxidant}}{\to }\;\mathrm{HCHO}$$

(4) $${\mathrm{CH}}_{4}\;+\;{\mathrm{CH}}_{3}\mathrm{OH}\;\overset{\mathrm{oxidant}}{\to }\;{\mathrm{CH}}_{3}{\mathrm{CH}}_{2}\mathrm{OH}\;\overset{\mathrm{oxidant}}{\to }\;{\mathrm{CH}}_{3}\mathrm{CHO}$$

(5) $${\mathrm{CH}}_{4}\;+\;\mathrm{HCHO}\;\overset{\mathrm{oxidant}}{\to }\;{\mathrm{CH}}_{3}\mathrm{CHO}$$

(6) $${\mathrm{CH}}_{3}\mathrm{OH}\;+\;\mathrm{HCHO}\;\overset{{}^{\mathrm{dehydration}}}{\to }\;{\mathrm{CH}}_{3}\mathrm{CHO}$$

In the primary reaction involved in CH_4_ oxidation, CH_4_ was oxidized by the oxidant (carbonate in this work) to form CH_3_OH, which was subsequently oxidized to HCHO. The production mechanism has been investigated in detail, and there are several ways to achieve the reaction.^36^ After that, several reactions can occur to generate acetaldehyde by employing the reactants CH_4_, methanol, and formaldehyde. Thus, the generation and accumulation of acetaldehyde is rapid and large, which is in accordance with the results in Table 1. For comprehensive understanding of the reaction processes, theoretical potentials of several oxidation reactions related to the methane conversion were listed in Table S2 (Supporting Information).

**Figure 5:**
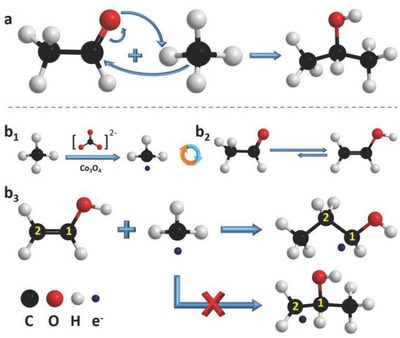
Reaction mechanism analysis. a) Nucleophilic addition reaction of methane and acetaldehyde to form 2‐propanol. b_1_–b_3_) Free radical addition reaction of methane and acetaldehyde to form 1‐propoanl.

The production of 1‐propanol and 2‐propanol from acetaldehyde is the most important reaction step in CH_4_ conversion. The mechanism involves a type of addition reaction, as shown in **Figure** **5**. The formation of 2‐propanol is common and has been reported in previous work.^23^ As shown in Figure 5a, the methyl group in CH_4_ acts as a nucleophilic reagent and attacks the carbonyl carbon in acetaldehyde. Then, a nucleophilic addition reaction occurs to form 2‐propanol as one of the main products in this CH_4_ conversion reaction. However, considering the addition reaction mechanism, the formation of 1‐propanol from acetaldehyde and CH_4_ is impossible. Therefore, we considered all the reaction conditions to determine a possible route for 1‐propanol production and found that the free radical addition reaction is suitable (Figure 5b1–b3). First, in route b1, a methyl radical is generated from CH_4_ with the participation of Co_3_O_4_ and carbonate. A carbonate radical is generated through anodic oxidation with the help of Co_3_O_4_ due to the relatively low generation energy compared with that of the hydroxyl radical,^37^ which can be obtained from electrochemical oxidation processes.^38^ The carbonate radical acted as an intermediate to generate a methyl radical through reaction with CH_4_. At the same time, in route b2, the as‐produced acetaldehyde equilibrates between its isomers, acetaldehyde, and vinyl alcohol. The vinyl alcohol configuration has a higher energy of 45 kJ mol^−1^ than the acetaldehyde form but is reachable in the presence of carbonate.^39^, ^40^ In the normal electrophilic addition to alkenes, the products follow the Markovnikov rule,^41^ illustrating 2‐propanol as the main product when CH_4_ reacts with vinyl alcohol. However, when the addition reaction is conducted through the free radical route, the products follow the anti‐Markovnikov rule, thereby producing 1‐propanol as the main product, as shown in route b3. When a methyl radical attacks carbon 1, the resulting 2‐propanol radical (free electron on carbon 2) is not the most stable state. However, when the methyl radical attacks carbon 2, the resulting 1‐propanol radical (free electron on carbon 1) is more stable than the 2‐propabol radical, illustrating 1‐propanol as the main product. 2‐Propanol can be directly converted from acetaldehyde and CH_4_ through a nucleophilic addition reaction, leading to more 2‐propanol being produced than 1‐propanol at short oxidation times. However, after long reaction times, the amount of 1‐propanol exceeds that of 2‐propanol, even though 2‐propanol is more thermodynamically stable, illustrating the unique regional selectivity of 1‐propanol production through radical addition with the participation of a Co_3_O_4_ catalyst and carbonate electrolyte. In summary, after comprehensive analysis with the above content, the complete reaction pathways for electrochemical oxidation of methane with ZrO_2_/Co_3_O_4_ anode and carbonate electrolyte were proposed, shown in Figure S12 (Supporting Information). The reaction process may help other researchers to have an overall understanding of the one carbon related reactions.

## 3 Conclusion

In summary, we designed a ZrO_2_/Co_3_O_4_ nanocomposite that aids in the regional selective oxidation of CH_4_ to 1‐propanol and 2‐propanol via an electrochemical method. We expected stable products, such as higher alcohols, to be formed due to the strong surface adsorption ability of Co_3_O_4_ and the participation of carbonate, which has a soft oxidizing ability, delivered by ZrO_2_. After tracking the products for different reaction times, acetaldehyde was found to be the key intermediate. To convert acetaldehyde to 1‐propanol with the participation of CH_4_, a free radical addition reaction was conducted by an electrochemical reaction, with Co_3_O_4_ as the catalyst and carbonate as the electrolyte. Finally, as a result of competition between reactions, both 1‐propanol and 2‐propanol were the main products. This electrochemical partial oxidation of CH_4_ may aid in the synthesis of other oxygenates and long‐chain hydrocarbons.

## 4 Experimental Section

*Fabrication of Electrocatalyst Materials*: All reagents were used as received without further treatment. The ZrO_2_/Co_3_O_4_ nanocomposite was synthesized using precipitation and a hydrothermal method. For the 1–2 ZrO_2_/Co_3_O_4_ sample, 0.1611 g ZrOCl_2_·8H_2_O (99.0%, Junsei, Japan), 0.291 g Co(NO_3_)_2_·6H_2_O (98%, Aldrich, US), and 9.6 g NaOH (96%, Samchun, Korea) were dissolved in 40 mL deionized (DI) water with vigorous stirring for 30 min. Then, the solution was transferred to a 60 mL autoclave container and heated at 180 °C for 24 h. After that, the powder was collected by centrifuge and washed with DI water 3 times. Finally, the 1–2 ZrO_2_/Co_3_O_4_ sample was obtained after thermal annealing at 500 °C for 3 h. For the ZrO_2_/Co_3_O_4_ nanocomposites with different ratios, the amount of Co(NO_3_)_2_·6H_2_O was adjusted to 0.582 g for 1–4 ZrO_2_/Co_3_O_4_ and 0.873 g for 1–6 ZrO_2_/Co_3_O_4_, with no changes in the other conditions. For comparison, pure Co_3_O_4_ and ZrO_2_/NiO samples were prepared by the same method as 1–4 ZrO_2_/Co_3_O_4_, without the addition of ZrOCl_2_·8H_2_O and with the addition of 0.582 g Ni(NO_3_)_2_·6H_2_O (97%, Aldrich, US) instead of Co(NO_3_)_2_·6H_2_O, respectively.

*Electrochemical Test*: LSV and EIS tests for the comparison of ZrO_2_/Co_3_O_4_ and ZrO_2_/NiO samples were conducted in a three‐electrode system using a potentiostat (CH Instrument, CHI 660) with a glassy carbon electrode as the working electrode, Ag/AgCl as the reference electrode, a Pt foil as the counter electrode and 0.5 m Na_2_CO_3_ solution as the electrolyte. Meanwhile, LSV tests for ZrO_2_/Co_3_O_4_ samples with different component ratio were conducted in a two‐electrode system without the utilization of Ag/AgCl reference electrode. All working electrodes were prepared by dispersing the powder samples in DI water in a concentration of 3 mg mL^−1^ with vigorous stirring for 30 min and then dropping 20 µL of the dispersed solution on the surface of the glassy carbon electrode (area = 0.07 cm^2^), followed by drying at room temperature. Next, 10 µL of a 5% Nafion 117 solution (Aldrich) was deposited on the surface of the glassy carbon electrode to cover the sample films, followed by drying at room temperature. Ultrahigh‐purity argon gas (Ar, 99.999%) and methane gas (CH_4_, 99.999%) were used. Before each electrochemical test, the electrolyte was bubbled with Ar or CH_4_ for 1 h to prepare the Ar‐ or CH_4_‐saturated electrolyte.

*CH_4_ Conversion Test*: The long‐term electrochemical oxidation of CH_4_ was conducted in a two‐electrode system with a closed reaction instrument, employing carbon paper (Alfa) as the working electrode, Pt foil as the counter electrode and 30 mL 0.5 m Na_2_CO_3_ solution (pH around 12.0 before reaction and about 11.9 after 12 h reaction) as the electrolyte. The working electrode was prepared by dispersing the powder sample in DI water in a concentration of 3 mg mL^−1^ with vigorous stirring for 30 min and then dropping 5.7 mL of the dispersed solution on the surface of the carbon paper (area = 20 cm^2^), followed by drying at room temperature. Next, 3 mL of a 5% Nafion 117 solution was deposited to cover the sample film on the carbon paper, followed by drying at room temperature. Before the electrochemical reaction, the electrolyte was bubbled with CH_4_ for 1.5 h to remove the oxygen and fill the space in the reaction instrument. In this case, after the consumption of saturated CH_4_ in aqueous solution, the gas phase CH_4_ could dissolve in the electrolyte continually, guaranteeing the adequate reactant. Electrochemical oxidation was conducted at 2.0 V versus Pt for 3, 6, or 12 h.

*Characterization and Products Analysis*: Morphology analyses of the samples were carried out using field‐emission scanning electron microscopy (FESEM, JSM‐7000F, Japan) and a JEOL JEM‐2100F (Japan) electron microscope. EDS spectra and ICP‐OES measurements were employed for the elements ratio detection. XRD measurements were conducted using a Siemens diffractometer D500/5000 in a Bragg–Brentano geometry. XPS data were obtained from a K‐alpha instrument (Thermo Scientific Inc., UK). ^1^H‐NMR was conducted using an Avance III HD 400 FT‐NMR instrument (Bruker Biospin), where the sample was prepared by mixing 0.4 mL of the product solution with 0.2 mL D_2_O. The amount of methane was determined using a 7890B GC instrument (Agilent Technologies). The product was examined using a 7890B‐5977A GC‐MS instrument (Agilent Technologies).

## Conflict of Interest

The authors declare no conflict of interest.

## Supporting information

Supplementary

## References

[1] Key World Energy Statistics 2015. International Energy Agency, Paris.

[2] Carbon Dioxide Emissions Coefficients by Fuel. U.S. Energy Information Administration, 2016.

[3] R. A. Kerr , Science 2010, 328, 1624. 20576864 10.1126/science.328.5986.1624

[4] A. R. Brandt , G. A. Heath , E. A. Kort , F. O. Sullivan , G. Petron , S. M. Jordaan , P. Tans , J. Wilcox , A. M. Gopstein , D. Arent , S. Wofsy , N. J. Brown , R. Bradley , G. D. Stucky , D. Eardley , R. Harriss , Science 2014, 343, 733. 24531957 10.1126/science.1247045

[5] D. T. Shindell , G. Faluvegi , D. M. Koch , G. A. Schmidt , N. Unger , S. E. Bauer , Science 2009, 326, 716. 19900930 10.1126/science.1174760

[6] R. J. Farrauto , Science 2012, 337, 659. 22879495 10.1126/science.1226310

[7] R. A. Alvarez , S. W. Pacala , J. J. Winebrake , W. L. Chameides , S. P. Hamburg , Proc. Natl. Acad. Sci. USA 2012, 109, 6435. 22493226 10.1073/pnas.1202407109 PMC3340093

[8] X. Chen , Y. Li , X. Pan , D. Cortie , X. Huang , Z. Yi , Nat. Commun. 2016, 7, 12273. 27435112 10.1038/ncomms12273 PMC4961799

[9] M. Cargnello , J. J. Delgado Jaen , J. C. Hernandez Garrido , K. Bakhmutsky , T. Montini , J. J. Calvino Gamez , R. J. Gorte , P. Fornasiero , Science 2012, 337, 713. 22879514 10.1126/science.1222887

[10] F. F. Tao , J. Shan , L. Nguyen , Z. Wang , S. Zhang , L. Zhang , Z. Wu , W. Huang , S. Zeng , P. Hu , Nat. Commun. 2015, 6, 7798. 26239771 10.1038/ncomms8798

[11] L. Yuliati , H. Yoshida , Chem. Soc. Rev. 2008, 37, 1592. 18648684 10.1039/b710575b

[12] Z. He , Q. Qian , J. Ma , Q. Meng , H. Zhou , J. Song , Z. Liu , B. Han , Angew. Chem., Int. Ed. 2016, 55, 737. 10.1002/anie.201507585 26602993

[13] V. C.‐C. Wang , S. Maji , P. P.‐Y. Chen , H. K. Lee , S. S.‐F. Yu , S. I. Chan , Chem. Rev. 2017, 117, 8574. 28206744 10.1021/acs.chemrev.6b00624

[14] P. Tang , Q. Zhu , Z. Wu , D. Ma , Energy Environ. Sci. 2014, 7, 2580.

[15] L. Nazaries , J. C. Murrell , P. Millard , L. Baggs , B. K. Singh , Environ. Microbiol. 2013, 15, 2395. 23718889 10.1111/1462-2920.12149

[16] G. A. Olah , A. Goeppert , M. Czaun , G. K. S. Prakash , J. Am. Chem. Soc. 2013, 135, 648. 23256664 10.1021/ja311796n

[17] I. M. Petrushina , V. A. Bandur , N. J. Bjerrum , F. Cappeln , L. Qingfeng , J. Electrochem. Soc. 2002, 149, D143.

[18] D. Eng , M. Stoukides , Catal. Rev. Sci. Eng. 1991, 33, 375.

[19] P. Promoppatum , V. Viswanathan , ACS Sustainable Chem. Eng. 2016, 4, 1736.

[20] Y. Amenomiya , V. Birss , M. Goledzinowski , J. Galuszka , A. R. Sanger , Catal. Rev. Sci. Eng. 1990, 32, 163.

[21] N. Spinner , J. A. Vega , W. E. Mustain , Catal. Sci. Technol. 2012, 2, 19.

[22] N. Spinner , W. E. Mustain , Electrochim. Acta 2011, 56, 5656.

[23] N. Spinner , W. E. Mustain , J. Electrochem. Soc. 2012, 159, E187.

[24] H. Ahn , T. J. Marks , J. Am. Chem. Soc. 1998, 120, 13533.

[25] B. Samaranch , P. Piscina , G. Glet , M. Houalla , P. Gelin , N. Homs , Chem. Mater. 2007, 19, 1445.

[26] N. Spinner , W. E. Mustain , J. Electrochem. Soc. 2013, 160, F1275.

[27] Z. Zhu , G. Lu , Z. Zhang , Y. Guo , Y. Guo , Y. Wang , ACS Catal. 2013, 3, 1154.

[28] J. Y. Kim , N. Choi , H. J. Park , J. Kim , D. Lee , H. Song , J. Phys. Chem. C 2014, 118, 25994.

[29] A. Peterson , F. Abild‐Pedersen , F. Studt , J. Rossmeisl , J. Norskov , Energy Environ. Sci. 2010, 3, 1311.

[30] T. Li , J. He , B. Pena , C. P. Berlinguette , Angew. Chem., Int. Ed. 2016, 55, 1769. 10.1002/anie.201509567 26689617

[31] M. Huang , Y. Zhang , F. Li , Z. Wang , Alamusi, N. Hu , Z. Wen , Q. Liu , Sci. Rep. 2014, 4, 4518. 24682149 10.1038/srep04518 PMC3970130

[32] J. Schattka , D. Shchukin , J. Jia , M. Antonietti , R. Caruso , Chem. Mater. 2002, 14, 5103.

[33] F. A. Carey , Organic Chemistry, 4th Ed., McGraw‐Hill Higher Education, Boston, MA, USA 2000, Ch. 13.

[34] Biological Magnetic Resonance Data Bank, The Board of Regents of the University of Wisconsin System.

[35] I. Bar‐Nahum , A. M. Khenkin , R. Neumann , J. Am. Chem. Soc. 2004, 126, 10236. 15315423 10.1021/ja0493547

[36] T. Iwasita , Electrochim. Acta 2002, 47, 3663.

[37] D. B. Medinas , G. Cerchiaro , D. F. Trindade , O. Augusto , IUBMB Life 2007, 59, 255. 17505962 10.1080/15216540701230511

[38] J. M. Kesselman , O. Weres , N. S. Lewis , M. R. Hoffmann , J. Phys. Chem. B 1997, 101, 2637.

[39] W. J. Bouma , L. Radom , W. R. Rodwell , Theor. Chim. Acta 1980, 56, 149.

[40] A. Karton , Chem. Phys. Lett. 2014, 592, 330.

[41] G. Jones , J. Chem. Educ. 1961, 38, 297.

