# Supplementary material for: Ultrahigh Electrocatalytic Conversion of Methane at Room Temperature
Source: Adv Sci (Weinh). 2017 Sep 11;4(12):1700379. doi: 10.1002/advs.201700379 (PMC5737395; doi:10.1002/advs.201700379)
Supplement: Supplementary file 1 — Supplementary [file ADVS-4-na-s001.pdf]

Copyright WILEY-VCH Verlag GmbH & Co. KGaA, 69469 Weinheim, Germany, 2017.

ADVANCED SCIENCE
Open Access

Supporting Information

for *Adv. Sci.,* DOI: 10.1002/advs.201700379

Ultrahigh Electrocatalytic Conversion of Methane at Room Temperature

*Ming Ma, Bing Jun Jin, Ping Li, Myung Sun Jung, Jin Il Kim, Yoonjun Cho, Sungsoon Kim, Jun Hyuk Moon, and Jong Hyeok Park**

Copyright WILEY-VCH Verlag GmbH & Co. KGaA, 69469 Weinheim, Germany, 2016.

Supporting Information

Ultra-high Electro-catalytic Conversion of Methane at Room Temperature

*Ming Ma, Bing Jun Jin, Ping Li, Myung Sun Jung, Jin Il Kim, Yoonjun Cho, Sungsoon Kim, Jun Hyuk Moon, and Jong Hyeok Park**

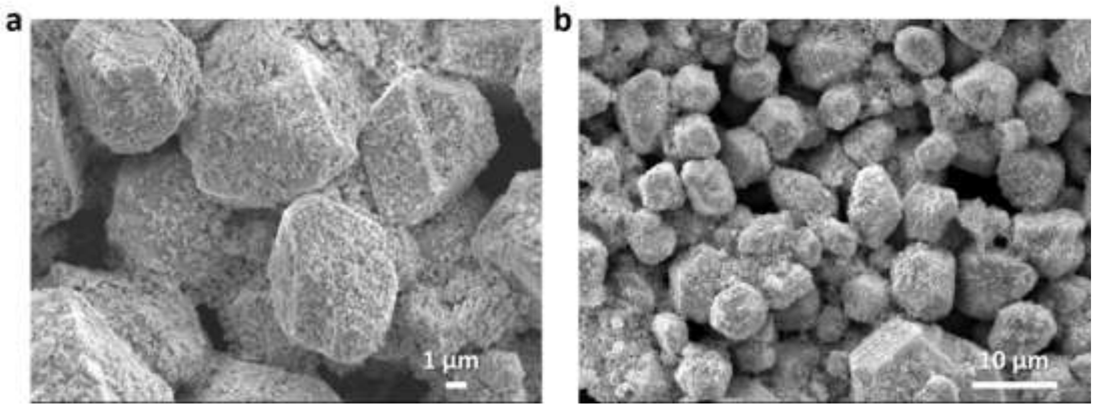

Figure S1. SEM images of the 1-6 ZrO_2/Co_3O_4 nanocomposite.

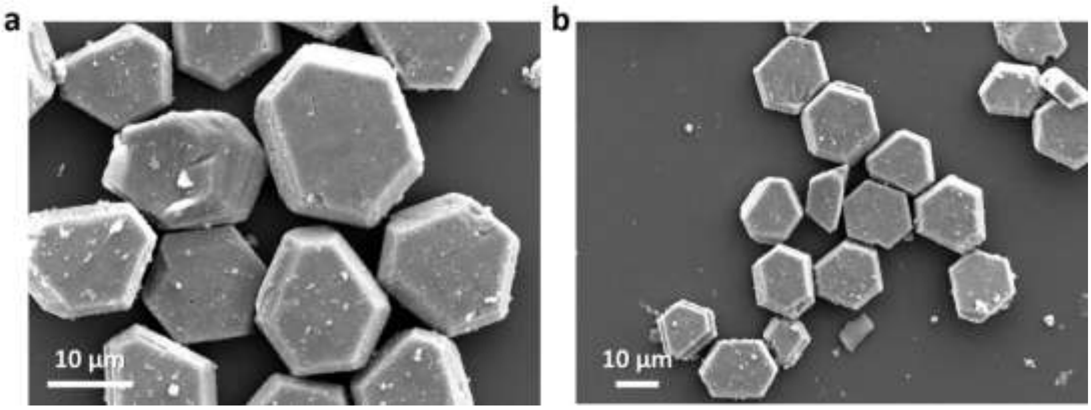

Figure S2. SEM images of pure Co_3O_4.

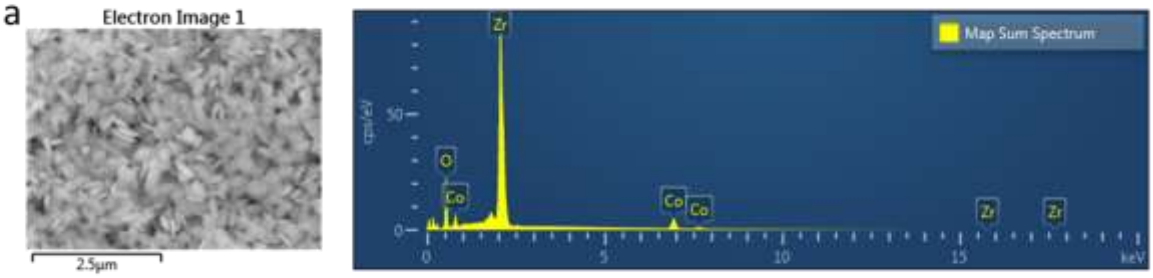

Element	Wt%	Wt% Sigma	Atomic %
O	23.13	0.17	61.29
Co	11.72	0.15	8.43
Zr	65.15	0.19	30.28
Total:	100.00		100.00

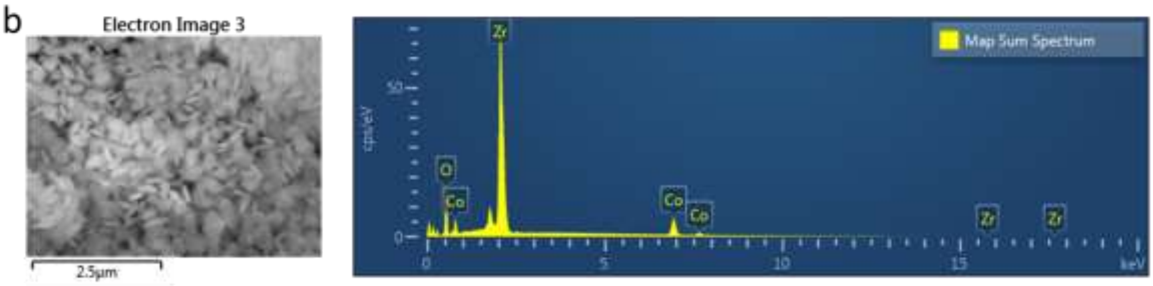

Element	Wt%	Wt% Sigma	Atomic %
O	19.85	0.20	55.70
Co	17.97	0.22	13.69
Zr	62.18	0.25	30.61
Total:	100.00		100.00

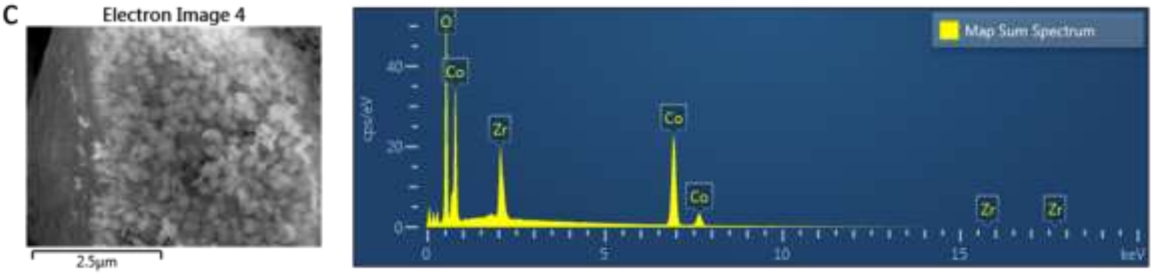

Element	Wt%	Wt% Sigma	Atomic %
O	25.09	0.28	57.02
Co	60.10	0.37	37.08
Zr	14.81	0.32	5.90
Total:	100.00		100.00

Figure S3. EDS spectra of the whole area shown in corresponding SEM images with elemental ratios for different samples as (a) 1-2 ZrO_2/Co_3O_4, (b) 1-4 ZrO_2/Co_3O_4 and (c)1-6 ZrO_2/Co_3O_4.

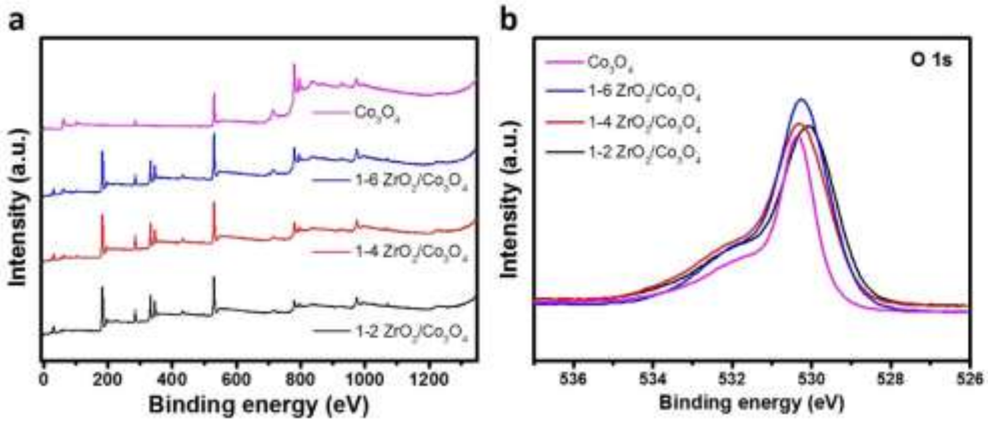

Figure S4. Characteristics of ZrO_2/Co_3O_4 nanocomposites with different ratios. (a) XPS survey and (b) overlapping O 1s XPS signals of pure Co_3O_4 and ZrO_2/Co_3O_4 with different ratios of 1-2, 1-4 and 1-6.

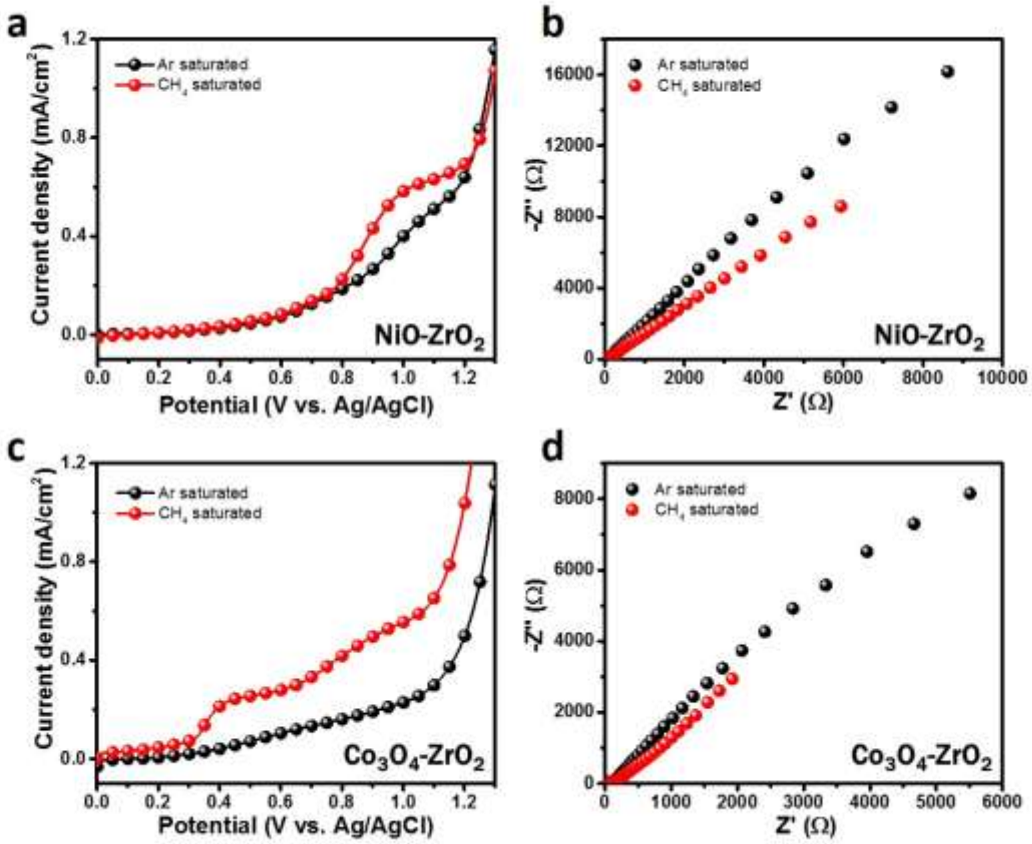

Figure S5. Electrochemical performance of ZrO_2/NiO and ZrO_2/Co_3O_4. LSV curves of (a) ZrO_2/NiO and (c) ZrO_2/Co_3O_4. EIS curves of (b) ZrO_2/NiO and (d) ZrO_2/Co_3O_4. The electrochemical impedance spectra (EIS) were measured in potentiostatic mode with an AC voltage amplitude of 5 mV over a frequency range of 0.1 - 100 kHz.

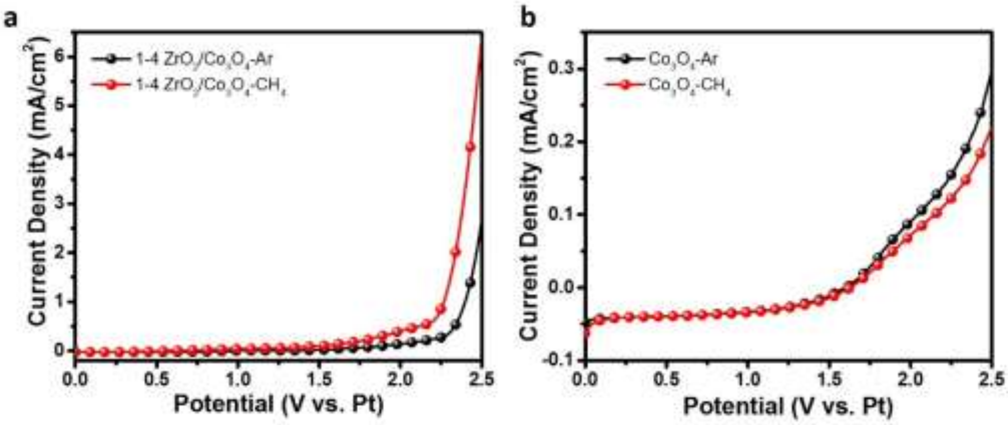

Figure S6. Electrochemical performance of 1-4 ZrO_2/Co_3O_4 and pure Co_3O_4. LSV curves of (a) 1-4 ZrO_2/Co_3O_4 and (b) Co_3O_4 in Ar- and CH_4-saturated electrolyte.

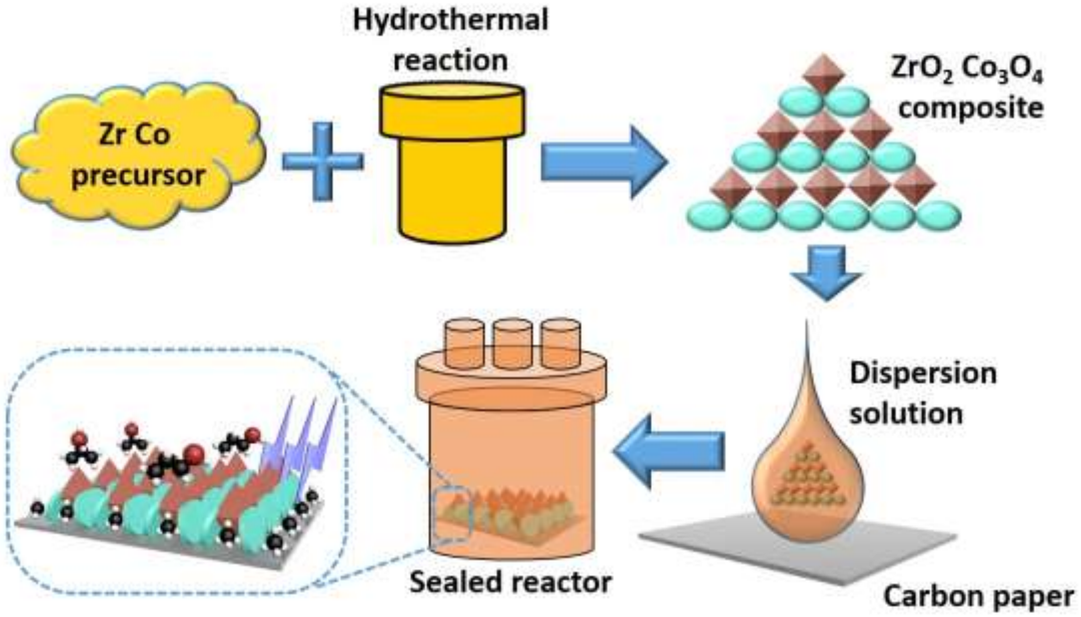

Figure S7. Schematic of the fabrication of the ZrO_2/Co_3O_4 nanocomposite and the electrochemical oxidation reaction with the prepared working electrode.

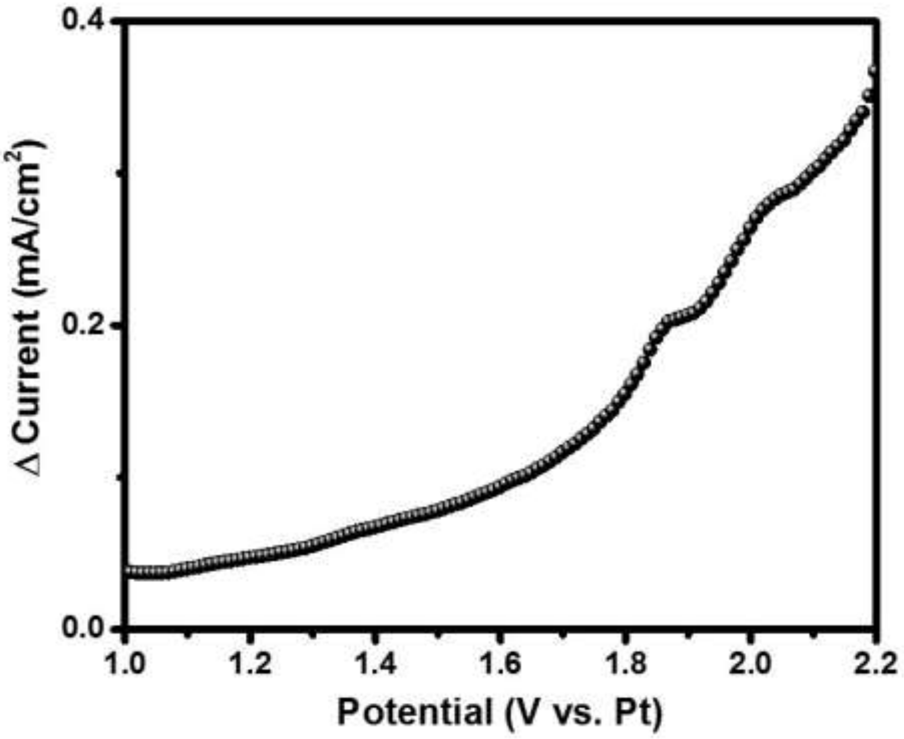

Figure S8. Difference in the current density of the 1-4 ZrO_2/Co_3O_4 sample in Ar- and CH_4-saturated electrolyte.

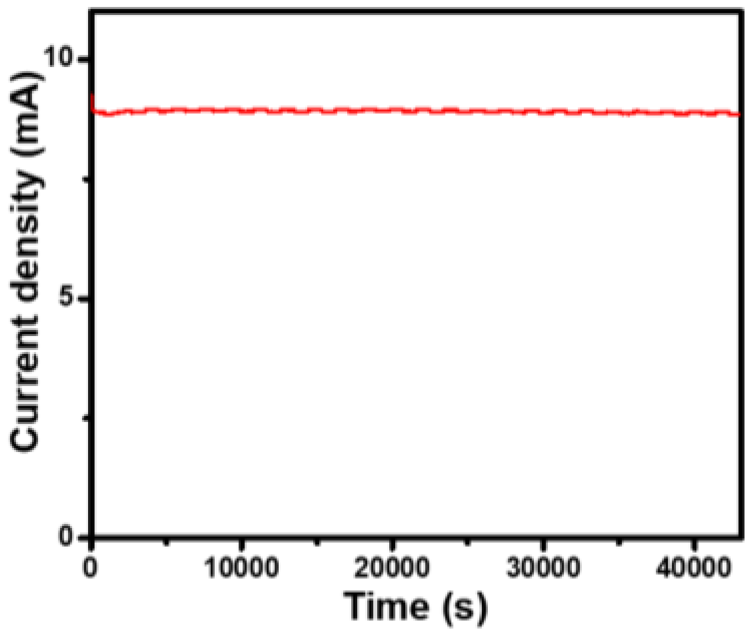

Figure S9. I-t curve of 1-4 ZrO_2/Co_3O_4 sample for long term electrochemical CH_4 oxidation reaction.

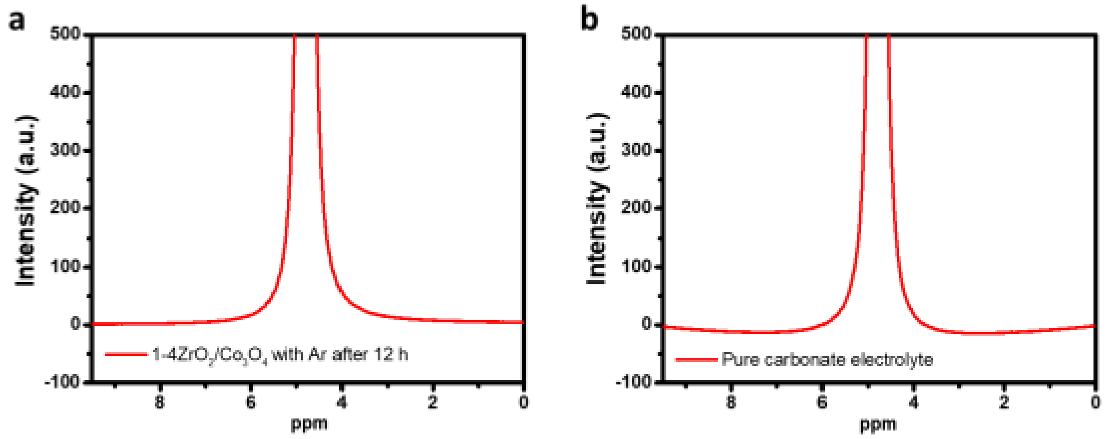

Figure S10. ^{1}H-NMR spectra of (a) 1-4 ZrO_2/Co_3O_4 sample with Ar after 12 h long-term reaction at 2.0 V vs. Pt and (b) pure carbonate electrolyte before reaction.

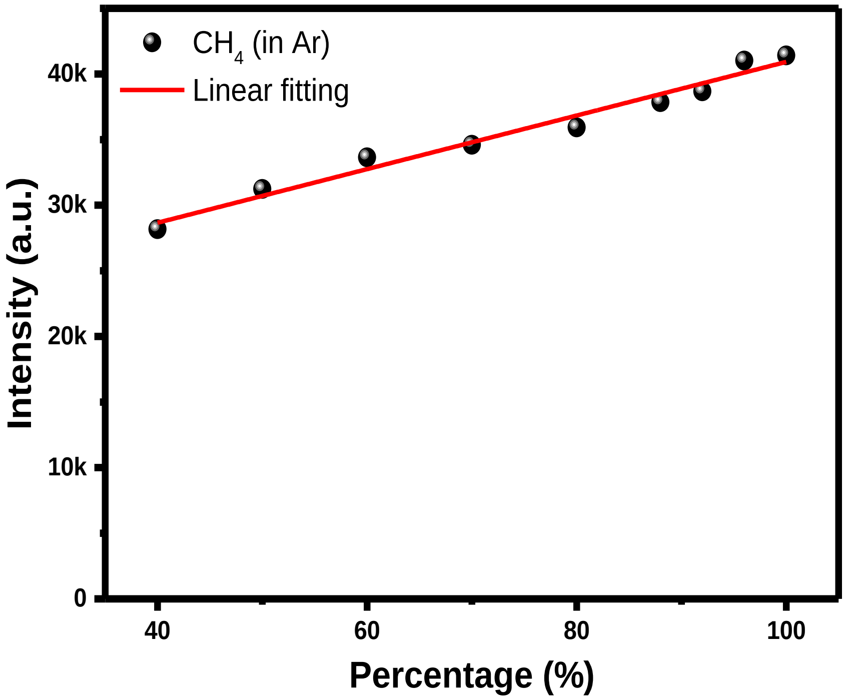

Figure S11. Reference line of the amount of CH_4 measured by GC.

$$(1)\ CH_4 + CO_3^{2-} + 2H_2O \rightarrow CH_3OH + CO_2 + H_2 + 2OH^-$$

$$(2)\ CH_3OH + CO_3^{2-} + H_2O \rightarrow HCHO + CO_2 + H_2 + 2OH^-$$

$$(3)\ HCHO + CH_4 + CO_3^{2-} + H_2O \rightarrow CH_3CHO + CO_2 + H_2 + 2OH^-$$

$$(4)\ CH_3CHO + CH_4 \rightarrow C_3H_8O$$

Figure S12. Complete reaction equations proposed for the electrochemical oxidation of methane.

Table S1. Elemental analysis of ZrO_2/Co_3O_4 nanocomposites with different ratios of 1-2, 1-4 and 1-6 from EDS spectra and ICP-OES measurements.

ZrO_2/Co_3O_4	Ratio of Zr/Co	
	EDS spectra	ICP-OES
1-2	3.59	0.515
1-4	2.24	0.240
1-6	0.16	0.163

Table S2. Theoretical potentials of several oxidation reactions related to the methane conversion.

Redox reaction (298.15 K, vs. RHE)			E^o (V)
CO_2 (g) + 2H^+ + 2e^-	⇌	HCOOH (aq)	−0.11
HCOOH (aq) + 2H^+ + 2e^-	⇌	HCHO (aq) + H_2O	−0.03
2H^+ + 2e^-	⇌	H_2 (g)	0.00
HCHO (aq) + 2H^+ + 2e^-	⇌	CH_3OH (aq)	+0.13
CH_3OH (aq) + 2H^+ + 2e^-	⇌	CH_4 (g) + H_2O	+0.50
O_2(g) + 4H^+ + 4e^-	⇌	2H_2O	+1.229
